# Supplementary figures and images for: Genetic screen identified PRMT5 as a neuroprotection target against cerebral ischemia
Source: eLife. 2024 Feb 19;12:RP89754. doi: 10.7554/eLife.89754 (PMC10942588; doi:10.7554/eLife.89754)

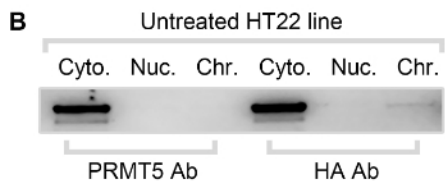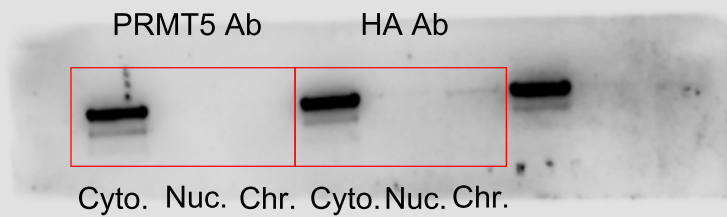

Supplement: Figure 2—source data 1. [file elife-89754-fig2-data1.zip › Figure 2-source data 1 (band labelled).pdf]

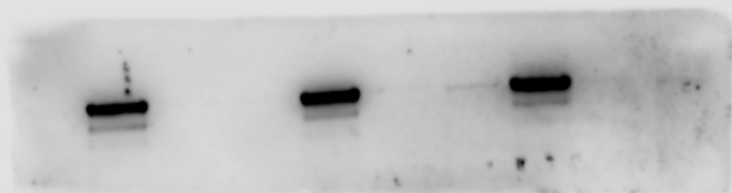

Supplement: Figure 2—source data 1. [file elife-89754-fig2-data1.zip › Figure 2-source data 1 (original file).pdf]

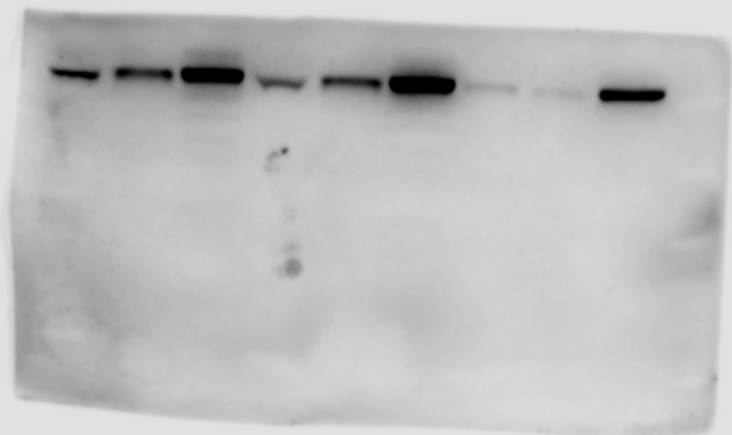

Supplement: Figure 2—source data 2. [file elife-89754-fig2-data2.zip › Figure 2-source data 2 (original file).pdf]

**B**

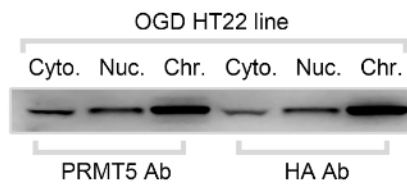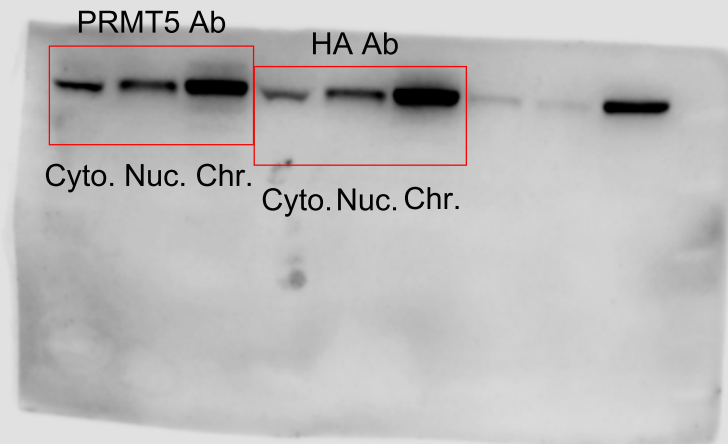

Supplement: Figure 2—source data 2. [file elife-89754-fig2-data2.zip › Figure 2-source data 2 (band labelled).pdf]

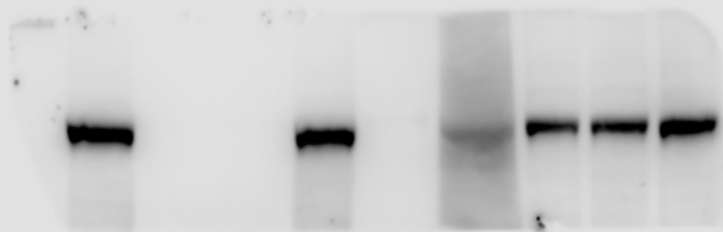

Supplement: Figure 2—source data 3. [file elife-89754-fig2-data3.zip › Figure 2-source data 3 (original file).pdf]

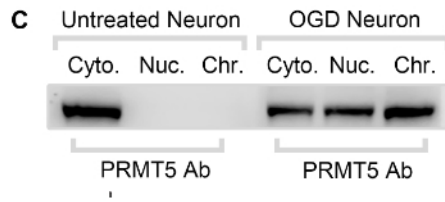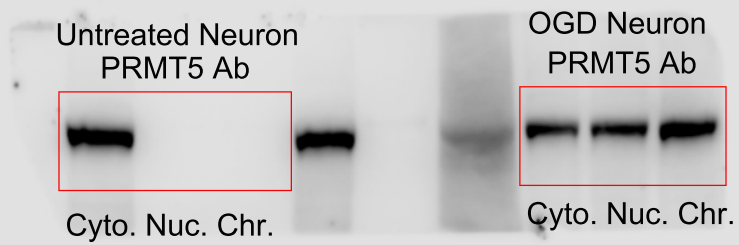

Supplement: Figure 2—source data 3. [file elife-89754-fig2-data3.zip › Figure 2-source data 3 (band labelled).pdf]

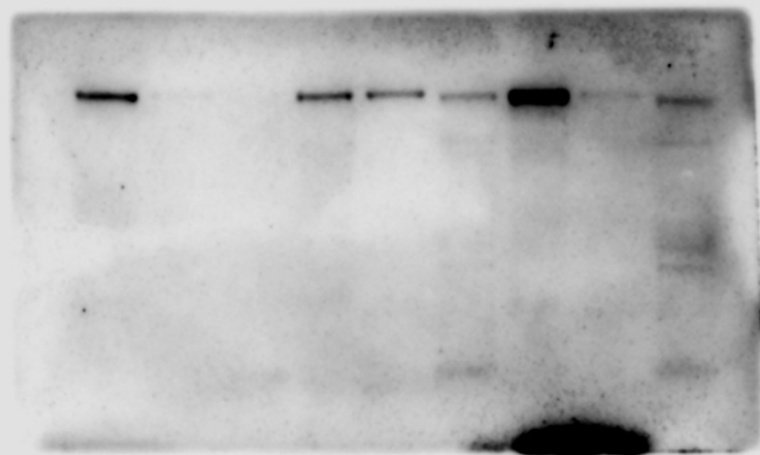

Supplement: Figure 3—source data 1. [file elife-89754-fig3-data1.zip › Figure 3-source data 1 (original file).pdf]

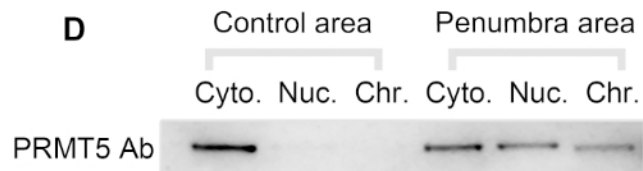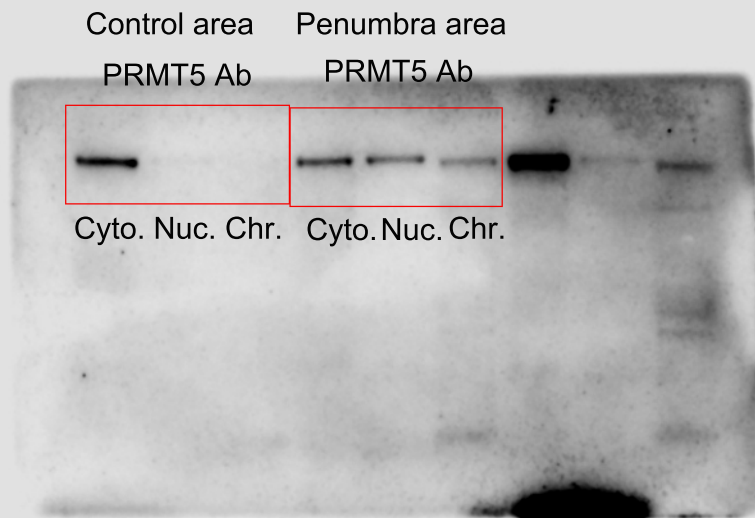

Supplement: Figure 3—source data 1. [file elife-89754-fig3-data1.zip › Figure 3-source data 1 (band labelled).pdf]
